# Unveiling the Artificial Intelligence (AI) Shift: Attitudes Toward and Utilization of AI Models Among Undergraduate Medical Students

**DOI:** 10.7759/cureus.110458

**Published:** 2026-06-08

**Authors:** Hitarthi M Joshi, Apexa B Shukla, Pavan J Panchal

**Affiliations:** 1 Pharmacology, GMERS Medical College and General Hospital, Himmatnagar, IND

**Keywords:** artificial intelligence tools, attitude, medical undergraduate students, perception, utilization

## Abstract

Introduction: Artificial intelligence (AI) tools like ChatGPT (OpenAI, San Francisco, USA) are increasingly influencing medical education, yet their use among undergraduate students in Western India remains underexplored. This study aimed to assess students’ acceptance, usage patterns, and key factors influencing AI adoption to support effective curriculum integration.

Methodology: This cross-sectional, questionnaire-based study evaluated attitudes toward and use of AI among undergraduate medical students from first year to final year. A structured 37-item English questionnaire was developed via Google Forms (Google LLC, Mountain View, USA) and distributed online using electronic channels, with informed consent obtained. Data were managed in Microsoft Excel 2013 (Microsoft Corp., Redmond, USA) and analyzed using GraphPad Prism version 10.6 (GraphPad Software, Boston, USA; www.graphpad.com), yielding key insights into perceptions and use of AI.

Results: This study was conducted among 791 medical students, with a mean age of 19.92 ± 1.64 years. Of these, 701 (88.62%) participants engaged with AI, relying predominantly on social media (425 (53.72%)) and faculty lectures (199 (25.15%)) for information. Moreover, 784 (99.11%) participants showed high AI awareness. Multivariable logistic regression demonstrated that AI use was strongly associated with behavior (odds ratio (OR)=356.9; 95% confidence interval (CI): 77.83-6329), perceived usefulness (OR=18.5; 95% CI: 40.68-327), and inversely with perceived risk (OR=0.06; 95% CI: 0.003-0.31). Students more than 20 years of age, male participants, and consistent AI users showed significantly higher usefulness and strong positive attitudes. Notably, first-year students reported a higher perception of risk compared to their peers, underscoring significant variations in AI adoption and perception.

Conclusions: The use of AI models among Western Indian undergraduate medical students and their attitudes were investigated in this study. Although there was variation in students' comprehension of AI-related concepts, the majority of students reported being aware of and having previously used AI tools, mostly through social media platforms. The use of AI and opinions of AI models were correlated with age, gender, academic year, perceived utility, and behavior. The results demonstrate the potential value of responsible and ethical AI guidance in medical education and may offer initial institutional-level insights for future multicentric research and curriculum development.

## Introduction

Artificial Intelligence (AI) can be defined as "the ability of machines to mimic intelligent human behavior, including problem-solving and learning" [1]. The rapid advancement of AI and large language models (LLMs), such as ChatGPT (OpenAI, San Francisco, USA), Google Bard (Google LLC, Mountain View, USA), and Microsoft Copilot (Microsoft Corp., Redmond, USA), has brought transformative changes across multiple sectors, including healthcare and medical education [2]. These AI tools offer significant potential in assisting healthcare professionals by enhancing diagnostic speed, accuracy, and treatment effectiveness across various medical fields, including clinical decision support [3]. Beyond this, LLMs are increasingly being used for tasks such as creating patient summaries, responding to clinical questions, and assisting with evidence-based practice in addition to direct clinical use.

Despite these developments, the use of AI in undergraduate medical education is still uneven and understudied. Undergraduate medical education represents a critical phase in the development of future healthcare practitioners. It is during this formative period that students acquire the foundational knowledge, skills, and attitudes that will shape their professional careers. A multinational study (2025) across seven countries found that 52% of medical students supported using LLMs in healthcare, although it focused on attitudes rather than actual usage frequency [4]. Therefore, curriculum designers and medical educators must comprehend how medical students view, utilize, and engage with AI tools.

Although teacher-led education remains an important component of the medical curriculum, student-led learning methods are gaining popularity due to their flexibility and superior engagement [5,6]. Among these, self-directed learning is an integral part of competency-based medical education. AI models can have a significant impact on self-directed or student-led learning due to their transformative potential in empowering learners to take control of their educational journey [7]. In an era where medical information is abundantly available but often overwhelming, LLMs provide students with a versatile tool to independently navigate, synthesize, and apply knowledge. However, the successful integration of AI models into medical education necessitates a comprehensive understanding of how these technologies are perceived and utilized by the very individuals they are designed to serve - undergraduate medical students [8].

While global studies have examined AI adoption in healthcare, region-specific insights - particularly within the Indian context - remain scarce. In Western India, encompassing states like Maharashtra, Gujarat, and Goa, medical colleges and hospitals are gradually integrating AI-based tools into academic and clinical settings. Nevertheless, there is limited empirical data on how undergraduate medical students in this region perceive and utilize these technologies [4,9,10]. Understanding their attitudes, utilization patterns, and apprehensions, including concerns about reliability, academic integrity, data privacy, and over-reliance on AI for clinical reasoning, is crucial for optimizing AI’s role in medical education. Such region-specific evidence can support curriculum planners and institutions in assessing students’ readiness for AI-assisted learning and identifying the support systems required [11,12].

Therefore, this study aimed to (i) assess awareness and utilization patterns of AI tools among undergraduate medical students in Western India; (ii) evaluate students’ perceptions regarding usefulness, risk, and attitudes toward AI; and (iii) identify demographic and behavioral predictors associated with AI adoption. Consequently, the findings of this study may provide valuable insights that can inform evidence-based strategies for integrating AI into undergraduate medical curricula effectively and responsibly.

## Materials and methods

Study design and setting

This study employed an observational, cross-sectional, questionnaire-based design to investigate the attitudes toward and utilization of AI models among undergraduate medical students at GMERS Medical College, Himmatnagar, located approximately 30 km from Idar Hill Fort and 50 km from the well-known pilgrimage site Shamlaji Temple in Western India. The study was conducted over a duration of three months (October 2025 to December 2025) after obtaining the required approval from the Institutional Ethics Committee (Approval No. GMERS/MCG/IEC/80/01/09/2025 dated 19/08/2025).

Sample and procedure

The target study population comprised undergraduate medical students (from the first year to the third final year) enrolled across four academic years. With an official intake of 200 students per year, approximately 800 eligible medical students were approached for participation during the study period. Participants were required to meet specific inclusion criteria: they had to be undergraduate medical students, willing to provide written informed consent, and possess an active WhatsApp, Telegram, or email account. Individuals were excluded from the study if they belonged to the administrative staff, were unwilling to participate, or provided incomplete data.

Development of the questionnaire

An anonymously filled questionnaire was constructed. The majority of questions were prepared from a previously published study from abroad to enhance the quality and validity of the data [13]. The original conceptual framework of the referenced questionnaire was retained.

For validation, the questionnaire was reviewed by two subject experts, who evaluated the time required for completion and ensured that all questions and sections were phrased clearly, appropriately, and without bias. Based on the feedback obtained during the expert validation process, necessary changes were incorporated. The questionnaire was subsequently pilot-tested on a small group of 20 medical students to assess comprehensibility, feasibility, and alignment with the study objectives. After pilot testing, minor language-related modifications were made, and these participants were not included in the final analysis. Formal reliability assessment, such as Cronbach's alpha, was not carried out in this study because the questionnaire items were derived from a previously published study with established conceptual frameworks. Before administering the questionnaire to the final study population, content validity was addressed through expert review and pilot testing.

Questions and scale

The questionnaire was prepared in the English language and comprised a total of 37 questions, systematically divided into several sections: five questions on general demographic information, five questions assessing knowledge related to AI models, six items on perceived usefulness, three items on behavioral aspects, five items on perception, five items on perceived risk, three items on anxiety factors, and five items addressing technology/social influence. Following the demographic and knowledge-related questions, the survey utilized conditional branching based on the response to the question: "Have you used any AI model (ChatGPT) before the study?" Participants reporting prior use of an AI model completed all remaining sections (27 items), whereas non-users completed only sections related to perceived risk, anxiety, and technology/social influence (13 items). The full questionnaire is provided in Appendix 1.

The questionnaire utilized multiple-choice questions along with a five-point Likert-type answer scale [13]. For scoring, "agree" corresponded to 5 points, "somewhat agree" to 4 points, "neutral/no opinion" to 3 points, "somewhat disagree" to 2 points, and "disagree" to 1 point. The total possible score ranged from 6 to 30 for perceived usefulness, with higher scores indicating greater perceived usefulness. Notably, the scoring was reversed for related items indicating a negative attitude to ensure consistency in interpretation. Composite scores were calculated by summing the responses of individual Likert-scale items within the relevant domains completed by each group, rather than throughout the entire questionnaire, owing to the differing number of applicable items between users and non-users. Consequently, domain-specific responses relevant to each participant group were used for comparative analyses, and no overall cumulative score combining the two groups was produced. For partially incomplete questionnaires, only completed responses were included in domain score calculations, and missing values were not imputed.

Data collection

Regarding data collection, an online questionnaire was developed using Google Forms (Google LLC, Mountain View, USA), configured to limit one-time participation per unique Internet Protocol (IP) address to prevent duplicate responses. The informed consent form and participant information sheet were embedded at the beginning of the questionnaire. After obtaining ethical approval, the questionnaire link was distributed to eligible participants through electronic messenger platforms such as WhatsApp, Telegram, Instagram, Facebook, email accounts, and other appropriate communication channels. Participation was entirely voluntary and without monetary compensation. At any point, participants could opt out of the survey or skip questions. No personally identifiable information was gathered to maintain anonymity and confidentiality. The survey was expected to take 10 minutes to complete, and participants were not followed up with. A total of 791 completed responses were received and included in the final analysis, resulting in a response rate of 98.8%. As convenience sampling was used, the possibility of selection bias cannot be completely excluded.

Statistical analysis

Data were retrieved from the online survey and saved in Microsoft Excel 2013 (Microsoft Corp., Redmond, USA). The collected data were then analyzed using GraphPad Prism version 10.6 for Windows (GraphPad Software, Boston, USA; www.graphpad.com).

Categorical data were presented as frequency (n) and percentage (%), whereas continuous data were presented as mean ± standard deviation (SD). The demographic comparisons between two independent groups based on age (≤20 vs. >20 years), gender (male vs. female), and prior AI use (yes vs. no) were analyzed using independent samples t-tests. A one-way analysis of variance (ANOVA) was performed to compare educational levels across four categories (first year, second year, third year - part I, and third year - part II), and the total p-value was provided. Binary logistic regression using maximum likelihood estimation was performed to find independent determinants of AI use among undergraduate medical students. AI usage was the dependent variable, whereas age, gender, behavior, and attitude were the independent variables selected based on the study objective, conceptual relevance from existing literature on technology acceptance and AI adoption in medical education, and the availability of variables within the structured questionnaire. Regression results were presented as regression coefficients (β), odds ratios (ORs), and their corresponding 95% confidence intervals (CIs), estimated using the profile likelihood method, along with associated p-values. Multicollinearity between independent variables was evaluated using the variance inflation factor (VIF) prior to model fitting. There was no significant multicollinearity among the predictors, as all VIF values were less than five (ranging approximately from 1.77 to 2.36). The following model assumptions were assessed: the study design (one response per participant) guaranteed observational independence, and the events-per-variable requirements for logistic regression indicated that the sample size was enough. Conceptually, linearity in the logit for continuous variables (age) was evaluated and found to be acceptable. There were no significant outliers that impacted model estimations. A complete-case analysis was carried out without imputation, as there were no missing values in the final dataset. Model goodness-of-fit was assessed using Tjur’s R^2 ^as a measure of explained variation. Likelihood ratio tests were also employed to assess the overall relevance of the model. When dummy-coding categorical data, third year I (profession) and female (gender) were used as reference categories. A p-value of ≤0.05 was considered statistically significant.

## Results

A total of 791 undergraduate medical students participated in this study. The mean age of the students was 19.92 ± 1.64 years, with a range of 18-25 years, and the median age was 20 years. Our study included 547 students (69.15%) aged 18-20 years, followed by 30.84% aged over 20 years. In terms of gender, 445 (56.25%) were male students and 346 (43.74%) were female students. Regarding the study year in the MBBS program, we enrolled 200 (25.28%) students from the first year, 208 (26.29%) from the second year, 197 (24.90%) from the third year I, and 186 (23.51%) from the third year II, as shown in Table 1.

**Table 1:** Demographic data AI: artificial intelligence

Demographic data		Frequency (n)	%
Age	18-20 years	547	69.15
	>20 years	244	30.84
Gender	Male	445	56.25
	Female	346	43.74
Profession	1^st^ year	200	25.28
	2^nd^ year	208	26.29
	3^rd^ year - part I	197	24.90
	3^rd^ year - part II	186	23.51
Have used AI	Yes	701	88.62
	No	90	11.37

**Figure 1:**
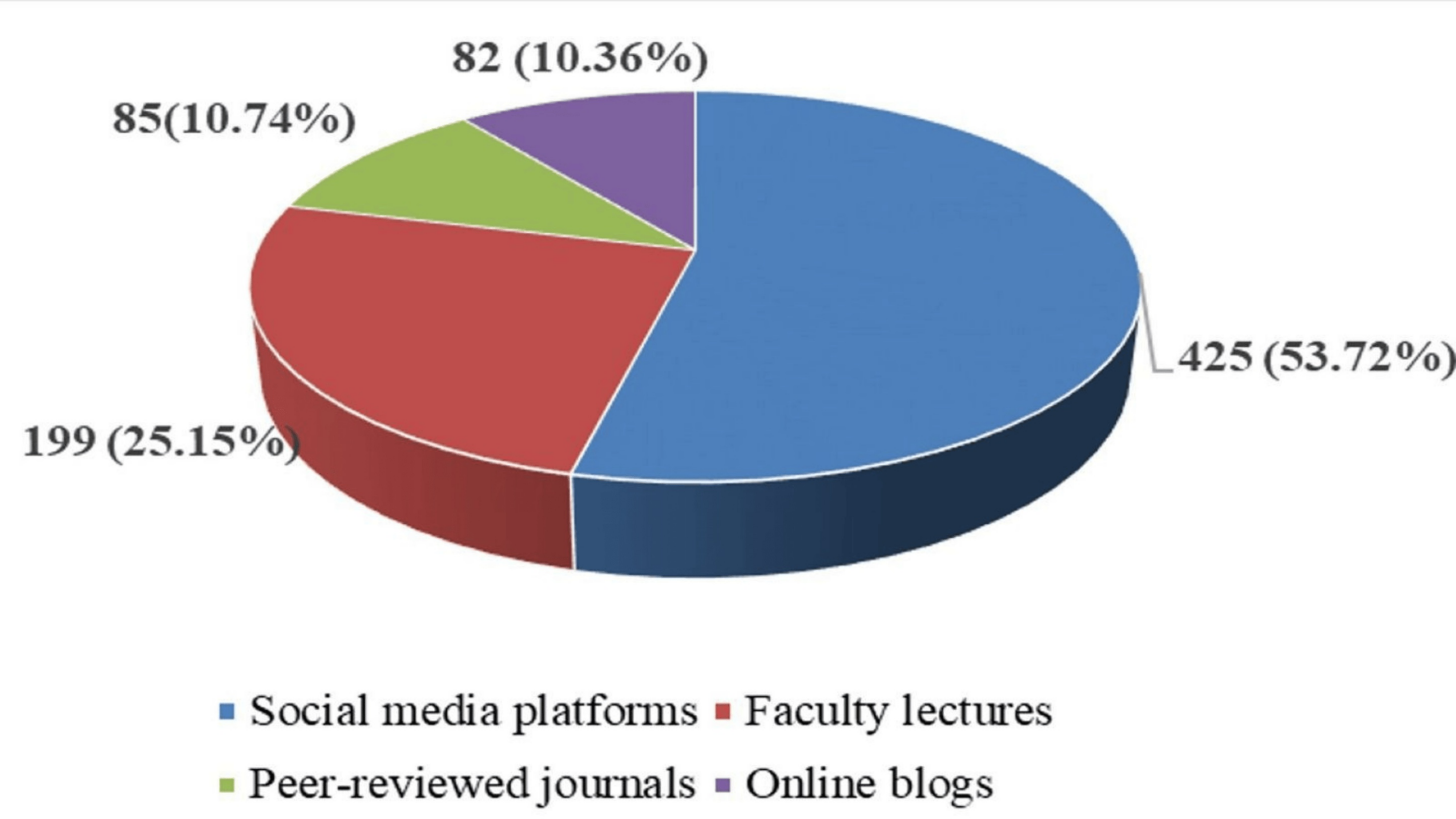
Sources used to learn AI among undergraduate medical students AI: artificial intelligence

In our study, we asked five questions about knowledge. Of these, one question focused on the abbreviation “AI,” while three questions were about examples of an AI model, a more accurate AI model, and a more consistent AI model. These were answered by 784 (99.11%), 556 (70.29%), 65 (8.21%), and 57 (7.20%) students, respectively. Social media platforms were identified as sources of AI information by 425 (53.72%) students, followed by 199 (25.15%) students who used faculty lectures as a source of information (Figure 1).

Relationship between AI use, demographic factors, perceived usefulness, perceived risk, and behavior

Multivariable logistic regression analysis (Table 2) showed that behavior was strongly associated with AI use (OR=356.9, 95% CI: 77.83-6329, p<0.001). Compared to the reference group, first-year students (OR=0.23, p=0.002) and third-year II students (OR=0.34, p=0.036) had significantly lower odds of perceiving AI use. Age, gender, and attitude were not significantly associated. AI use was strongly influenced by perceived usefulness (OR=18.5; 95% CI: 40.68-327, p<0.0001), while perception showed a strong and statistically significant inverse relationship (OR=0.06, 95% CI: 0.003-0.31, p<0.0001). We also found that second-year students had significantly higher odds of positive perception (OR=1.78; 95% CI: 1.03-3.11, p=0.039). In our study, perceived usefulness was strongly associated with lower risk (OR=0.009, 95% CI: 0.0005-0.041; p<0.0001). First-year students had significantly higher odds of risk compared to the reference group (OR=4.76, 95% CI: 1.66-15.37; p=0.003). Age, gender, and other academic years were not significantly associated with risk.

**Table 2:** Multivariable logistic regression model showing predictors of AI use among undergraduate medical students (n=791) *P-value ≤0.05 considered statistically significant. ^#^Reference group in profession: 3^rd ^year - part I. OR: odds ratio; AI: artificial intelligence; CI: confidence interval; β: regression coefficient

Variable	β	95% CI (profile likelihood)	OR	95% CI (profile likelihood)	P-value
Intercept	2.10	-3.52 to 7.71	8.16	0.02 to 22	0.46
Age (in years)	-0.04	-0.31 to 0.22	0.95	0.73 to 1.25	0.75
Profession^#^ (1^st^ year)	-1.47	-2.46 to -0.52	0.22	0.08 to 0.58	0.002*
Profession (2^nd^ year)	-0.23	-1.18 to 0.67	0.78	0.30 to 1.96	0.60
Profession (3^rd^ year - part II)	-1.07	-2.13 to -0.06	0.34	0.11 to 0.93	0.03*
Gender (male)	0.28	-0.28 to 0.84	1.32	0.75 to 2.33	0.32
Behavior	5.87	4.35 to 8.75	356.9	77.83 to 63	<0.0001*
Attitude	-0.21	-0.91 to 0.47	0.80	0.39 to 1.60	0.54
Tjur's R^2 ^= 0.34					

Differences in students’ perceived usefulness and attitudes according to demographic characteristics

As shown in Table 3, perceived usefulness toward AI among undergraduate medical students varied significantly with age >20 years, gender (male), and AI usage. Furthermore, Tukey’s comparison test applied to check variations between professional years of study showed statistically significant differences between the first and second years, and between the second and third years (p<0.001), as well as a statistically significant difference between the first and third year II (p<0.008). Table 4 indicates that students older than 20 years had a more positive attitude compared to other age groups (p<0.0001). Male students, third-year students, and AI users also exhibited a significantly more positive attitude compared to other groups.

**Table 3:** Differences in the students' perceived usefulness level according to demographic data T-test applied. *P-value ≤0.05 considered statistically significant. ^#^Indicates one-way analysis of variance (ANOVA) test. AI: artificial intelligence; SD: standard deviation; df: degrees of freedom

Demographic data		Mean	SD	t	Df	P-value
Age (in years)	≤20	4.14	0.32	-2.12	789	0.035*
	>20	4.19	0.30	-	-	-
Gender	Male	4.24	0.28	6.94	789	0.001*
	Female	4.08	0.35	-	-	-
Profession^#^	1^st^ year	4.25	0.28	-	-	0.0001*
	2^nd^ year	4.07	0.34	-	-	-
	3^rd^ year - part I	4.23	0.31	-	-	-
	3^rd^ year - part II	4.15	0.30	-	-	-
Have used AI	Yes	4.17	0.30	8.95	789	0.0001*
	No	3.52	0.68	-	-	-

**Table 4:** Differences in the students' attitude level according to demographic data T-test applied. *P-value ≤0.05 considered statistically significant. ^#^Indicates one-way analysis of variance (ANOVA) test. AI: artificial intelligence; SD: standard deviation; df: degrees of freedom

Demographic data		Mean	SD	t	df	P-value
Age (in years)	≤20	1.47	0.37	9.3	789	0.0001*
	>20	1.84	0.57	-	-	-
Gender	Male	1.70	0.48	5.76	789	0.001*
	Female	1.52	0.37	-	-	-
Profession^#^	1^st^ year	1.71	0.55	-	-	0.0001*
	2^nd^ year	1.48	0.43	-	-	-
	3^rd^ year - part I	1.23	0.15	-	-	-
	3^rd^ year - part II	2.00	0.63	-	-	-
Have used AI	Yes	1.71	0.41	3.03	789	0.003*
	No	1.75	0.51	-	-	-

## Discussion

This study explores the perspectives of undergraduate medical students from a single medical college in Western India regarding AI and their actual use of it. With 791 students providing input, we gained insight into both the excitement and the nervousness surrounding the integration of AI into medicine and education. We present the key findings and discuss their implications below.

First, almost all (88.62%) of these students had already used AI tools. Most of this exposure came from social media - over half mentioned it as their main source. The digital world clearly shapes future doctors’ knowledge of AI, often before they ever encounter it in a classroom. This is not unique to India; students everywhere seem to discover AI on their own, usually through informal channels such as blogs and social networks. Although it is great that knowledge is more accessible, there is a drawback: information from these sources is not always reliable [1,14]. Only about a quarter of the students said they learned about AI from faculty lectures, which highlights a gap: medical schools need to step up and ensure that students receive accurate, evidence-based information about AI.

However, when it comes to naming AI models or understanding their accuracy and consistency, the numbers drop sharply. Less than 10% could answer these deeper questions correctly. Students were using AI, but most lacked a deep understanding. Other studies support this - students are excited, but their knowledge is usually shallow [15]. That is risky, as using a tool without understanding it increases the risk of errors, particularly in healthcare.

Differences emerge when considering who feels what. First-year and third-year II students were less likely to see the value in using AI compared to second-year students, who appeared most positive about AI - perhaps because they were transitioning from theory to more hands-on material, where AI becomes genuinely useful. Gender also plays a role. Male students reported higher perceived usefulness and more positive attitudes than female students. According to similar findings observed in previous studies, male participants frequently indicated higher levels of confidence, familiarity, and readiness to experiment with developing digital technologies [15,16]. Additionally, older students (those over 20 years) tended to have more positive opinions of AI models. This could be linked to greater exposure to academic material, more opportunities for individual study, and increased use of digital learning tools during the later years of medical school.

The strongest predictor of AI use was perceived usefulness. If students saw the benefits, they worried less about risks such as accuracy, ethics, or over-reliance on machines. First-year students, who have had limited exposure, were the most concerned about risks. This underscores the importance of teaching AI basics early to build confidence and reduce perceptions of AI as risky.

Behavior also matters. Students who used AI tools were more likely to continue using them, consistent with global observations that students who take initiative with new technology persist in its use [16,17]. However, a positive attitude alone was insufficient; without hands-on experience, attitude did not reliably predict actual AI use. This is significant for educators: to encourage the adoption of new technology, providing opportunities for use is necessary rather than simply discussing it [18].

How this stacks up globally

These results align with global developments. Jackson et al. (2024) found that students in Europe have heard much about AI, but they lack understanding [1]. Das et al. (2023) showed ChatGPT can handle basic microbiology questions but struggles with complexity, raising concerns about over-reliance [16]. In India, Mondal et al. (2025) showed that AI research is booming, but a gap remains in clinical application [19]. When considered together, it is clear that we need to introduce AI into medical education in such a balanced way that students learn to question and evaluate what AI tells them - not simply accept it at face value [19,20]. Therefore, medical schools must step up and set clear guidelines for using AI and teach students how to think critically and act ethically with these tools [19].

What this means for medical education

This study highlights that AI literacy requires a permanent place in the medical curriculum. This means structured lessons on not only how AI works but also on ethics, legal issues, and how these tools fit into real clinical work. Teachers need support too because they are the ones helping students figure this out. Moreover, bringing in experts from computer science and engineering could provide medical students with a much better understanding of how AI actually works under the hood. It is not just about the technology; it is about seeing the bigger picture.

Limitations

There are several limitations that should be considered when interpreting the results of this study. The study only included participants from one medical college in Western India and employed a non-probability convenience sample technique, which may limit the findings' external validity and generalizability to other institutions or geographical regions. Second, since the study used self-reported responses, the results may be affected by recall bias, social desirability bias, and possible over-reporting of familiarity or utilization of AI tools. Third, causal relationships between variables could not be established due to the cross-sectional design. In addition, participants completed varying numbers of questionnaire items depending on their prior exposure to AI models, as the questionnaire employed conditional branching based on prior AI use. There was also some overlap between the perception and perceived risk domains due to the inclusion of conceptually related questions, which may have caused partial construct redundancy. Despite the fact that most of the questionnaire domains were obtained from a previously published study, formal psychometric validation techniques like Cronbach's alpha and factor analysis were not independently carried out for the adapted questionnaire domains in the current study. Finally, some regression estimates showed large CIs, which may indicate possible model instability or overfitting and necessitate careful interpretation of the regression results.

## Conclusions

This study explored the attitudes toward and utilization of AI models among undergraduate medical students at a medical college in Western India. Most students reported awareness and prior use of AI tools, primarily through social media platforms, although variability in understanding regarding AI-related concepts was observed. Factors such as age, gender, academic year, perceived usefulness, and behavior were found to be associated with AI utilization and perceptions of AI models among students. The findings highlight the importance of offering structured guidelines for the ethical and appropriate application of AI in medical education. The results may provide preliminary institutional-level insights for future curriculum planning, policy creation, and further multicentric research on AI use among medical students, even though educational interventions were not specifically addressed in this study.

## Appendices

Appendix 1 

**Table 5:** Questionnaire Each question was scored on a five-point Likert scale (agree, somewhat agree, neutral/ no opinion, somewhat disagree, disagree). *Two items (plagiarism concern and security risk concern) were intentionally repeated across perception and perceived risk domains to evaluate overlapping attitudes toward AI use. The questionnaire was adapted from Abdaljaleel et al. [13], licensed under a Creative Commons Attribution 4.0 International License (CC BY 4.0) (https://creativecommons.org/licenses/by/4.0/). The original 27 attitude items were retained with minor modifications. The knowledge-based questions were developed by the authors with the assistance of AI tools, including ChatGPT (OpenAI, San Francisco, USA), Gemini 2.5 (Google DeepMind, London, UK), Perplexity (Perplexity AI Inc., San Francisco, USA), and DeepSeek (Hangzhou DeepSeek Artificial Intelligence Basic Technology Research Co., Ltd., Hangzhou, China). AI: artificial intelligence

A. Demography and Knowledge	
1.	Participant's initials
2.	Age (in years)
3.	Profession (1^st^, 2^nd^, 3^rd^ part I, 3^rd^ part II MBBS year)
4.	Gender (male/female/other)
5.	Nationality (Indian/other)
6.	What does the abbreviation “AI” stand for? Artificial Innovation, Artificial Intelligence, Automated Interface, Algorithm Integration
7.	Which of the following is an example of an AI model? Gemini 2.5, ChatGPT, Perplexity, Deepseek, All of the above
8.	Which source do medical students/heathcare professionals most frequently use to learn about new AI models in medicine? Peer-reviewed journals, Social media platforms, Faculty lectures, Online blogs
9.	Which AI model is more accurate? ChatGPT 3.5, Grok, Gemini 2.5, Perplexity
10.	Which AI model gives more consistent results? ChatGPT 3.5, Grok, Gemini 2.5, Perplexity
Have you used any AI model (ChatGPT) before the study? Yes/No	
B. Percieved Usefulness	
11.	AI model helps me to save time when searching for information
12.	For me, AI model like ChatGPT is a reliable source of accurate information
13.	I recommend ChatGPT to my colleagues to facilitate their academic duties
14.	ChatGPT is more useful than other sources of information that I have used previously
15.	I appreciate the accuracy and reliability of the information provided by ChatGPT
16.	I believe that using ChatGPT can save time and effort in my college assignments and duties
C. Behavior/Cognitive Factors	
17.	I have used tools or techniques similar to ChatGPT in the past
18.	I spontaneously find myself using ChatGPT when I need information for my college assignments and duties
19.	I often use ChatGPT as a source of information in my college assignments and duties
D. Perception (Reversed Score)	
*20.	I am concerned that using ChatGPT would get me accused of plagiarism (Reversed Score)
*21.	I am concerned about the potential security risks of using ChatGPT (Reversed Score)
22.	I think that relying on technology like ChatGPT can disrupt my critical thinking skills (Reversed Score)
23.	It does not take a long time to learn how to use ChatGPT
24.	ChatGPT does not require extensive technical knowledge
E. Perceived Risk (Reversed Score)	
25.	I am concerned about the reliability of the information provided by ChatGPT (Reversed Score)
*26.	I am concerned that using ChatGPT would get me accused of plagiarism (Reversed Score)
*27.	I am concerned about the potential security risks of using ChatGPT (Reversed Score)
28.	I am afraid that the use of ChatGPT would be a violation of academic and college policies (Reversed Score)
29.	I am concerned about the potential privacy risks that might be associated with using ChatGPT (Reversed Score)
F. Anxiety (Reversed Score)	
30.	I am afraid of relying too much on ChatGPT and not developing my critical thinking skills (Reversed Score)
31.	I am afraid of becoming too dependent on technology like ChatGPT (Reversed Score)
32.	I am afraid that using ChatGPT would result in a lack of originality in my college assignments and duties (Reversed Score)
G. Technology/Social Influence	
33.	I am enthusiastic about using technology such as ChatGPT for learning and research
34.	I believe technology such as ChatGPT is an important tool for academic success
35.	I think that technology like ChatGPT is attractive and fun to use
36.	I am always keen to learn about new technologies like ChatGPT
37.	I trust the opinions of my friends or colleagues about using ChatGPT
